# Exosomes are involved in iron transport from human blood–brain barrier endothelial cells and are modified by endothelial cell iron status

**DOI:** 10.1016/j.jbc.2022.102868

**Published:** 2023-01-03

**Authors:** Kondaiah Palsa, Stephanie L. Baringer, Ganesh Shenoy, Vladimir S. Spiegelman, Ian A. Simpson, James R. Connor

**Affiliations:** 1 Department of Neurosurgery, Penn State College of Medicine, Hershey, Pennsylvania, USA; 2 Department of Pediatrics, Penn State College of Medicine, Hershey, Pennsylvania, USA; 3 Department of Neural and Behavioral Sciences, Penn State College of Medicine, Hershey, Pennsylvania, USA

**Keywords:** endothelial cells, exosomes, iron, transferrin, H-ferritin, BBBEC, blood–brain barrier endothelial cell, DFO, deferoxamine, EC, endothelial cell, EV, extracellular vesicle, FAC, ferric ammonium citrate, FPN1, ferroportin1, FTH1, H-ferritin, HEPH, hephaestin, hiPSC, human induced pluripotent stem cell, IRE, iron regulatory element, NTA, nanoparticle tracking analysis, TEER, transendothelial electrical resistance, TEM, Transmission electron microscopy, Tf, transferrin

## Abstract

Iron is essential for normal brain development and function. Hence, understanding the mechanisms of iron efflux at the blood–brain barrier and their regulation are critical for the establishment of brain iron homeostasis. Here, we have investigated the role of exosomes in mediating the transfer of H-ferritin (FTH1)- or transferrin (Tf)-bound iron across the blood–brain barrier endothelial cells (BBBECs). Our study used ECs derived from human-induced pluripotent stem cells that are grown in bicameral chambers. When cells were exposed to ^55^Fe-Tf or ^55^Fe-FTH1, the ^55^Fe activity in the exosome fraction in the basal chamber was significantly higher compared to the supernatant fraction. Furthermore, we determined that the release of endogenous Tf, FTH1, and exosome number is regulated by the iron concentration of the endothelial cells. Moreover, the release of exogenously added Tf or FTH1 to the basal side *via* exosomes was significantly higher when ECs were iron loaded compared to when they were iron deficient. The release of exosomes containing iron bound to Tf or FTH1 was independent of hepcidin regulation, indicating this mechanism by-passes a major iron regulatory pathway. A potent inhibitor of exosome formation, GW4869, reduced exosomes released from the ECs and also decreased the Tf- and FTH1-bound iron within the exosomes. Collectively, these results indicate that iron transport across the blood–brain barrier is mediated *via* the exosome pathway and is modified by the iron status of the ECs, providing evidence for a novel alternate mechanism of iron transport into the brain.

Iron is essential for brain development and its deficiency leads to impaired cognitive function and several neurological disorders (1, 2, 3). Conversely, excess iron can lead to free radical formation, oxidative stress, and neurodegeneration (4, 5). Thus, regulation of iron transport to the brain is essential. Earlier studies identified transferrin (Tf) as the major carrier protein responsible for the transport of iron to the brain (6, 7). Tf-bound iron is taken up *via* transferrin receptor (TfR) expressed on the luminal side of the blood–brain barrier endothelial cells (BBBECs), which leads to receptor-mediated endocytosis (8, 9), and studies have shown receptor-mediated transcytosis of Tf-bound iron directly from the luminal side of the BBBECs to the brain (10, 11). We have established that H-ferritin (FTH1) can also transport iron to the brain both *in vitro* and *in vivo* (12, 13). Ferritin is an iron storage protein capable of binding 4500 iron atoms, that is made up of two subunits: FTH1 and L-ferritin (14, 15). FTH1 has ferroxidase activity, which transforms ferrous iron into ferric iron (16). Our studies have shown that T-cell immunoglobulin and mucin domain1 is a receptor for FTH1 in BBBECs (12) and oligodendrocytes (17), and there are also reports that FTH1 will bind to the TfR1 (18, 19).

Brain iron uptake is mediated by BBBECs (20, 21, 22, 23, 24). The BBB is primarily composed of endothelial cells (ECs), pericytes, and astrocytes (25, 26). The astrocytes are in close contact with the blood vessels and previous studies have demonstrated that the release of iron from the BBBECs, *via* ferroportin1 (FPN1), is responsive to the astrocytic iron concentration that is mediated by hepcidin (9, 27). Hepcidin is a 3kDa peptide that binds to FPN1 and leads to its internalization and subsequent degradation (28, 29).

This study identifies a novel mechanism of Tf- and FTH1-bound iron transport across the blood–brain barrier that is mediated by exosomes. Extracellular vesicles (EVs) are secreted by all types of cells to the extracellular space and are also found in biological fluids. EVs are lipid-bound vesicles containing proteins, lipids, sugars, and nucleic acids which can cross cell membranes (30, 31). EVs are categorized into three types based on their size and origin. Exosomes that are 30 to 150 nm size are derived from intraluminal vesicles of late endosomes/multivesicular bodies that fuse with the plasma membrane and are released as exosomes. Microvessels and apoptotic bodies are nearly 0.1 to 1 and 5 μm size, respectively, and are released by the outward swelling and rupture of the plasma membrane (32, 33). In this study, we focused on the exosomes using the biomarkers CD63 and CD81, which are tetraspanin family members that are highly expressed on the surface of these intraluminal vesicles of late endosomes and are involved in the exosomes secretion (34).

The objective of our current study was to gain a better understanding of the pathway(s) of Tf- and FTH1-bound iron and the release of these proteins into the brain. We have investigated the role of BBBEC iron status on exosome release. We hypothesize that iron is released into the brain interstitium *via* exosomes and that the release of exosomes is modified by the iron status of the BBBECs.

## Results

### BBBECs release the exosome to the basal chamber

**Figure 1:**
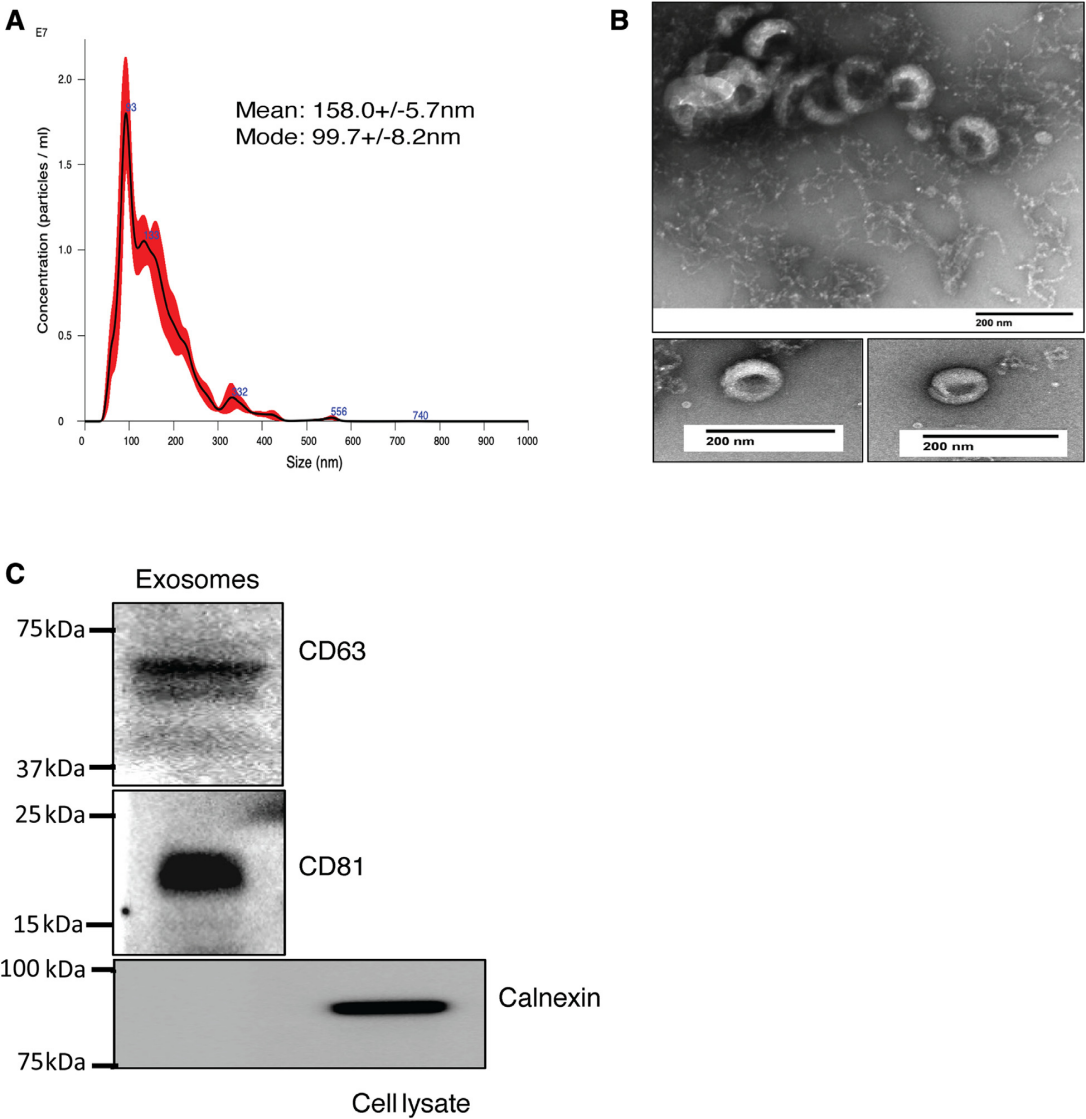
**BBBECs release the exosome to the basal chamber:** ECs were grown on 0.4 μm transwell plates and exosomes were isolated from the basal chamber media by using sequential ultracentrifugation. *A*, representative image of Nanoparticle tracking analysis of exosome sizes. *B*, transmission electron microscopy images of exosomes. Specimens were negatively stained with uranyl acetate the scale bar represents 200 nm for the images. *C*, immunoblot analysis of exosomes, which illustrates the characteristic exosome membrane proteins CD63 (40–63kDa), and CD81 (20–22kDa). Calnexin (90kDa) is a negative marker for exosomes, present in the endoplasmic reticulum membrane and was used here on cell lysates. BBBECs, blood–brain barrier endothelial cells; ECs, endothelial cells.

Our previous studies showed that Tf and FTH1 can transport iron to the brain *via* BBBECs with a maximal effect at 1 mg/ml and 100 μg/ml (23), respectively, hence all the experiments in the present study were performed with these concentrations. For all experiments, we used His-FTH1 (approximately 28 kD) which enabled us to distinguish experimental FTH1 from endogenous FTH1(21 kD) (Fig. S1). To determine whether Tf and FTH1 are released from the basal surface of human induced pluripotent stem cells (hiPSC)-derived BBBECs *via* exosomes, we first confirmed that exosomes were indeed being released from the basal side of ECs. This was achieved, as illustrated in Figure 1, by performing nanoparticle tracking analysis (NTA), transmission electron microscopy (TEM), and immunoblotting. NTA results demonstrated that most of the particles are approximately 100 nm in size but also contain some large size vesicles (Fig. 1*A*). TEM was performed after negative staining with uranyl acetate, and images demonstrated that the multiple particles are typical cup-shaped morphology and were approximately 100 nm (Fig. 1*B*). These results are consistent with the size of exosomes isolated from the cell culture media (35). Furthermore, immunoblotting of particles isolated by ultracentrifugation of the basal chamber media confirmed the expression of exosome surface protein markers CD63 and CD81 and further confirmed by negative control Calnexin, which is expressed in the endoplasmic reticulum membrane (Fig. 1*C*).

### BBBECs transport ^55^Fe-Tf and ^55^Fe-FTH1 *via* exosomes to the basal chamber

**Figure 2:**
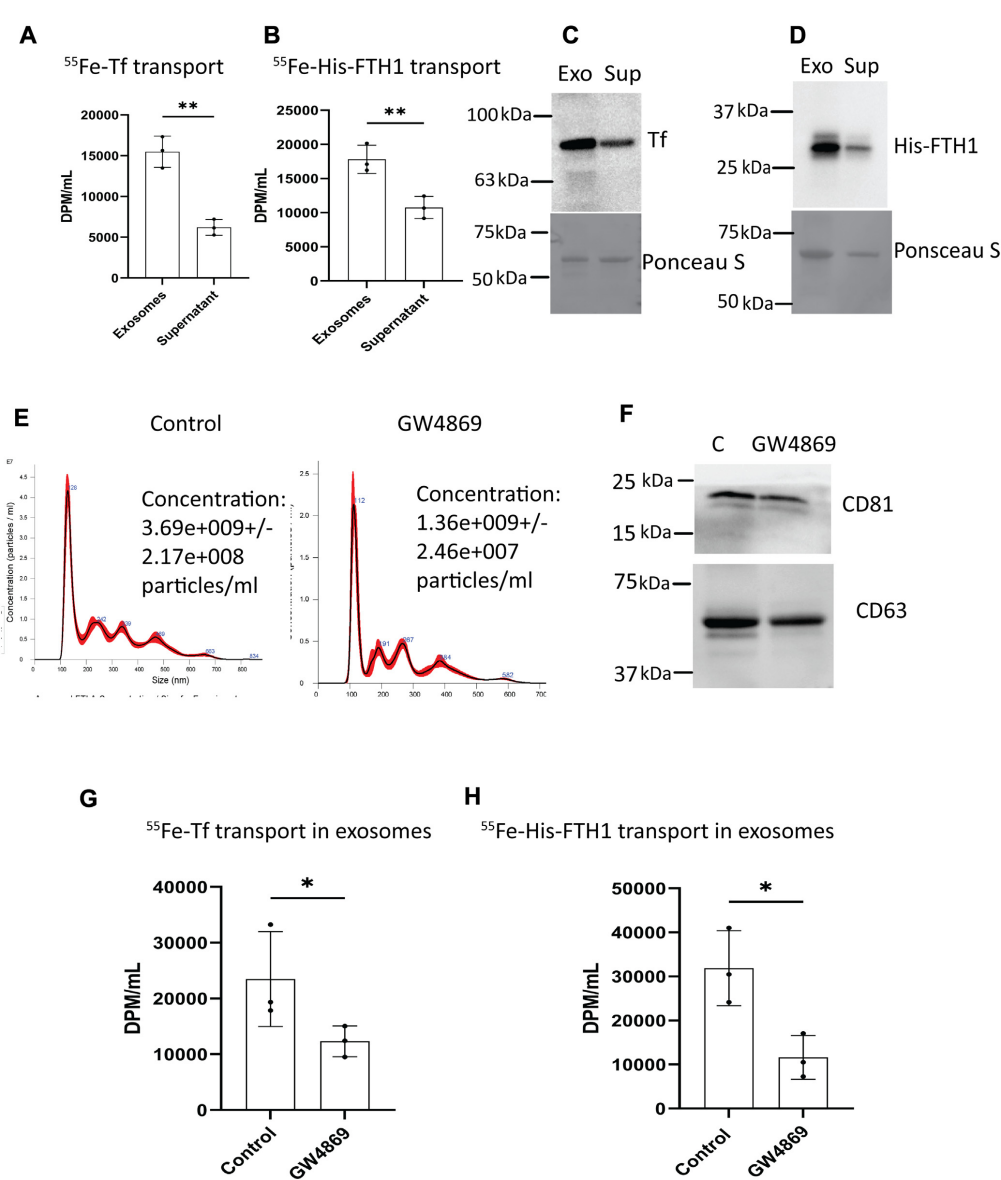
**BBBECs transport**^**55**^**Fe -Tf and**^**55**^**Fe -FTH1 *via* exosomes to the basal chamber.** ECs grown on the 0.4 μm pore size filters were incubated with GW4869 20 μM or 1 mg/ml of ^55^Fe-Tf or 100 μg/ml of ^55^Fe-FTH1 added to the apical chamber. After 24 h, exosomes were isolated from the basal chamber. The medium was measured for ^55^Fe activity in the exosomes *versus* supernatant fractions. ^55^Fe activity was measured in a liquid scintillation counter, results are expressed in DPM/ml. *A*, compares the ^55^Fe-Tf activity in the exosomes and supernatant fractions. The data shown are the mean ± S.D. of three independent replicates, Unpaired *t* test, ∗∗*p* < 0.01. *B*, compares the ^55^Fe-His-FTH1 activity in the exosomes and supernatant fractions. The data shown represent the mean ± S.D. of three independent replicates, Unpaired *t* test. ∗∗*p* < 0.01. *C* and *D*, demonstrate the corresponding immunoblot analysis for Tf and His-FTH1 in the respective exosomes (Exo) and supernatant (Sup) fractions. *E*, illustrates the exosome concentration released in control *versus* GW4869-treated cells. *F*, the immunoblots of CD81 and CD63 in control *versus* GW4869-treated cells. *G* and *H*, illustrate the exosomal ^55^Fe-Tf and ^55^Fe-His-FTH1 activity respectively in control *versus* GW4869-treated cells. The data shown are the mean ± S.D. of three independent replicates, Unpaired *t* test, ∗*p* < 0.05. All experiments were performed with three technical replicates in each biological replicate. BBBECs, blood–brain barrier endothelial cells; ECs, endothelial cells; FTH1, H-ferritin; Tf, transferrin.

Our previous studies demonstrated that hiPSC-derived BBBECs transport and release Tf- or FTH1-bound iron to the basal side (23).To determine whether Tf- or FTH1-bound iron is released from the BBB *via* exosomes, we radiolabeled Tf and His-FTH1 with ^55^Fe. The ^55^Fe-Tf and or ^55^Fe-His-FTH1 were incubated on the apical side of the ECs for 24 h, and activity was subsequently measured in exosomes isolated from the basal media. Radiolabeled iron from both Tf and His-FTH1 was isolated in the exosome fraction at approximately 2 to 3× the levels found in the supernatant (nonexosome containing) fraction (Fig. 2, *A* and *B*). In order to determine if our findings applied to Tf and His-FTH1 proteins and not just Fe, we added Tf or His-FTH1 into the apical chamber of the ECs cultures for 24 h. Exosomes were then isolated from the basal side and immunoblotted for Tf and His-FTH1. Immunoblotting demonstrated that both Tf and His-FTH1 were present in amounts 2× higher within the exosomes compared to the supernatant fraction, indicating that exosomes are the major route for transport of Tf and His-FTH1 across the BBB (Fig. 2, *C* and *D*). GW4869 is a potent inhibitor of sphingomyelinase which is essential for exosome formation (36). We first confirmed that GW4869 inhibited the release of exosomes in our BBBEC model by measuring the exosome concentration and membrane proteins (CD81, CD63) using NTA and immunoblotting, respectively (Fig. 2, *E* and *F*). We further studied whether the ^55^Fe-Tf or ^55^Fe-His-FTH1 transport to the basal chamber *via* exosomes was inhibited by GW4869. For this study, ECs were first treated with GW4869 (20 μM) for 2 h and then either ^55^Fe-Tf or ^55^Fe-His-FTH1 were added to the apical chamber and incubated ± inhibitor for 24 h. After 24 h, exosomes were isolated from the basal chamber media and measured for ^55^Fe activity. The ^55^Fe activity was significantly decreased in the GW4869-treated cells compared to the control for both ^55^Fe-Tf– and ^55^Fe-His-FTH1–treated cells (Fig. 2, *G* and *H*). These results further confirm that Tf- and FTH1-bound iron is transported through BBBECs *via* exosomes.

### BBBECs iron concentration regulates the cellular exosome membrane proteins

**Figure 3:**
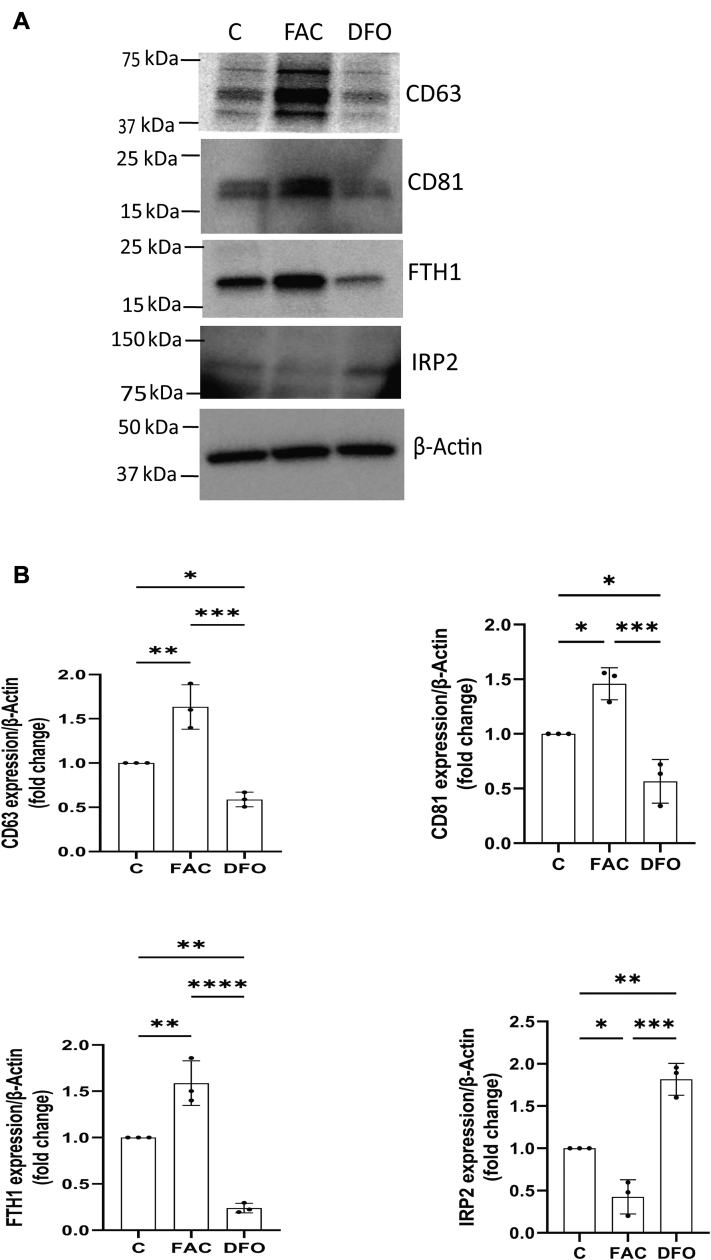
**BBBECs iron concentration regulates the cellular exosome membrane proteins.** ECs were grown on the 0.4 μm pore size filters and treated with ferric ammonium citrate (100 μmol/L) or DFO (100 μmol/L) for 12 h. *A*, representative cellular immunoblot analysis (MW in kDa shown in parentheses) of exosomes membrane proteins CD63 (40–63), CD81 (20–22), and iron responsive proteins FTH1 (20) and IRP2 (100). β-Actin (42) is present as the loading control. *B*, histograms for CD63, CD81, FTH1, and IRP2/β-Actin expressions are presented as fold change relative to the control (data have been normalized to the untreated control for each independent experiment), and the data are shown as the mean ± S.D. of three independent replicates and analyzed by one-way ANOVA with Tukey’s post-hoc test. ∗*p* < 0.05, ∗∗*p* < 0.01, ∗∗∗*p* < 0.001, ∗∗∗∗*p* < 0.0001. All experiments were performed with three technical replicates in each biological replicate. BBBECs, blood–brain barrier endothelial cells; DFO, deferoxamine; ECs, endothelial cells; FAC, ferric ammonium citrate; FTH1, H-ferritin; IRP2, iron regulatory protein 2.

The intracellular iron concentration regulates levels of ferritin (H and L chains) (37) and the exosomes membrane protein (CD63) through the iron regulatory elements (IREs) in the corresponding mRNA (38). To determine whether the iron concentration of the BBBECs alters the expression of cellular exosome membrane proteins, we treated ECs with 100 μmol/L of ferric ammonium citrate (FAC) or deferoxamine (DFO, an iron chelator) for 12 h. The FAC treatment increased the expression of CD63, CD81, and FTH1 expression (Fig. 3, *A* and *B*) whereas their expression was decreased by DFO when compared to the control. The expression of the iron regulatory protein 2 (IRP2), which binds to the IREs, was decreased by FAC loading and increased by DFO, thus supporting its regulatory role in the expression of CD63 and CD81. These data indicated that intracellular iron concentration regulates exosome membrane proteins.

### BBBECs iron concentration alters the release of exosomes and exosome containing FTH1 and Tf into the basal chamber

**Figure 4:**
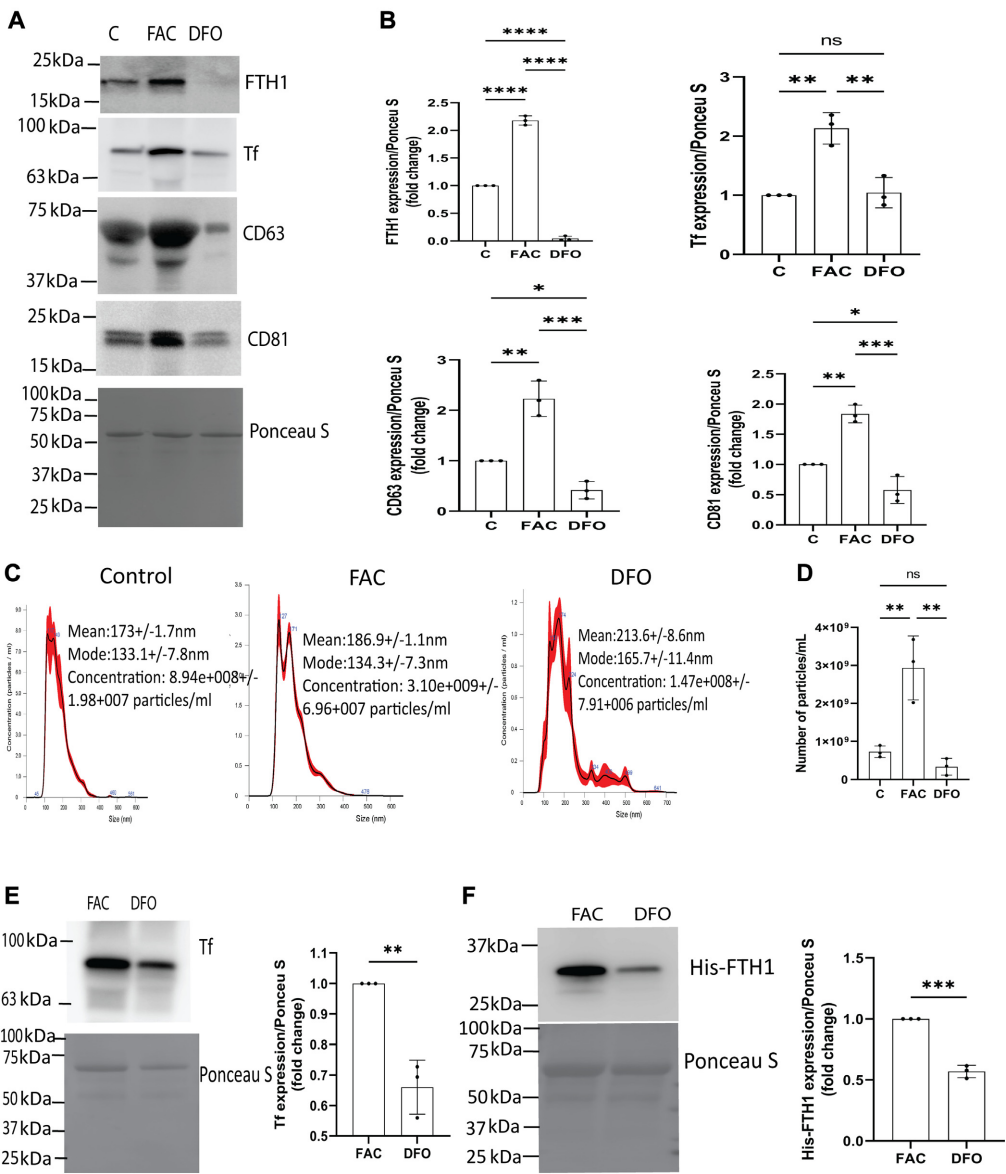
**BBBECs iron concentration alters the release of exosomes and exosomes containing FTH1 and Tf into the basal chamber.** ECs were grown on the 0.4 μm pore size filters treated with ferric ammonium citrate (FAC, 100 μmol/L) or deferoxamine (DFO, 100 μmol/L) for 24 to 48 h, the basal media were collected and the exosomes isolated. This experiment was designed to demonstrate exosomes release, and the Tf and FTH1 content of the exosomes were impacted by the BBBECs iron status. *A*, illustrates the immunoblot analysis of exosomes for detection of FTH1, transferrin, CD63, and CD81 expression. *B*, the corresponding graphical analysis of FTH1, transferrin, CD63, and CD81/ponceau S expressions are presented as fold change relative to the control (data have been normalized to the untreated control for each independent experiment). The data shown represent the mean ± S.D. of three independent replicates and analyzed by one way-ANOVA, ∗*p* < 0.05, ∗∗*p* < 0.01, ∗∗∗*p* < 0.001, ∗∗∗∗*p* < 0.0001. *C* and *D*, the exosomes concentration was measured by nanoparticle tracking analysis (NTA), and data were expressed as particles/ml. The data shown represent the mean ± S.D. of three independent experiments and analyzed by one way-ANOVA, ∗∗*p* < 0.01; *E* and *F*, ECs were treated with 100 μmol/L FAC or DFO, and after 24 h, His-FTH1 or Tf were added to the apical chamber and incubated 24 h. This experiment was designed to demonstrate that exogenously applied His-FTH1 was transcytosed *via* exosomes isolated from the basal chamber, and the amount of His-FTH1 found in the exosomes was altered by the iron status of the cells. The levels of Tf in the exosomes of the basal chamber were also altered by FAC and DFO treatment. *E* and *F* illustrates representative immunoblots of Tf and His-FTH1 in exosomes respectively. His-FTH1 and Tf/ponceau S expressions are presented as fold change relative to FAC (data have been normalized to the FAC treatment for each independent experiment). The data shown represent the mean ± S.D. of three independent replicates and analyzed by unpaired *t* test, ∗∗*p* < 0.01, ∗∗∗*p* < 0.001. All experiments were performed with three technical replicates in each biological replicate. BBBECs, blood–brain barrier endothelial cells; ECs, endothelial cells; FTH1, H-ferritin; Tf, transferrin.

To assess whether the iron concentration of ECs impacts the release of exosomes and their levels of Tf or FTH1, we treated ECs with 100 μmol/L FAC or DFO for 48 h and isolated exosomes from the basal chamber media. The exosomes from FAC-treated cells released significantly higher levels of FTH1, Tf, CD63, and CD81 compared to the control and DFO treatment (Fig. 4, *A* and *B*). We further studied the impact of iron loading ECs with FAC on exosome release. FAC treatment of ECs significantly increased the release of exosomes relative to DFO or control-treated groups (Fig. 4, *C* and *D*). We further studied the transport of exogenous Tf or His-FTH1 in FAC- or DFO-treated ECs. The ECs were pretreated with FAC or DFO for 24 h followed by subsequent exposure to Tf or His-FTH1. FAC-treated ECs had significantly higher levels of Tf and His-FTH1 in the released exosomes compared to DFO-treated ECs (Fig. 4, *E* and *F*).

### The release of exosomes containing ^55^Fe-Tf or ^55^Fe-His-FTH1 is independent of hepcidin regulation in BBBECs

**Figure 5:**
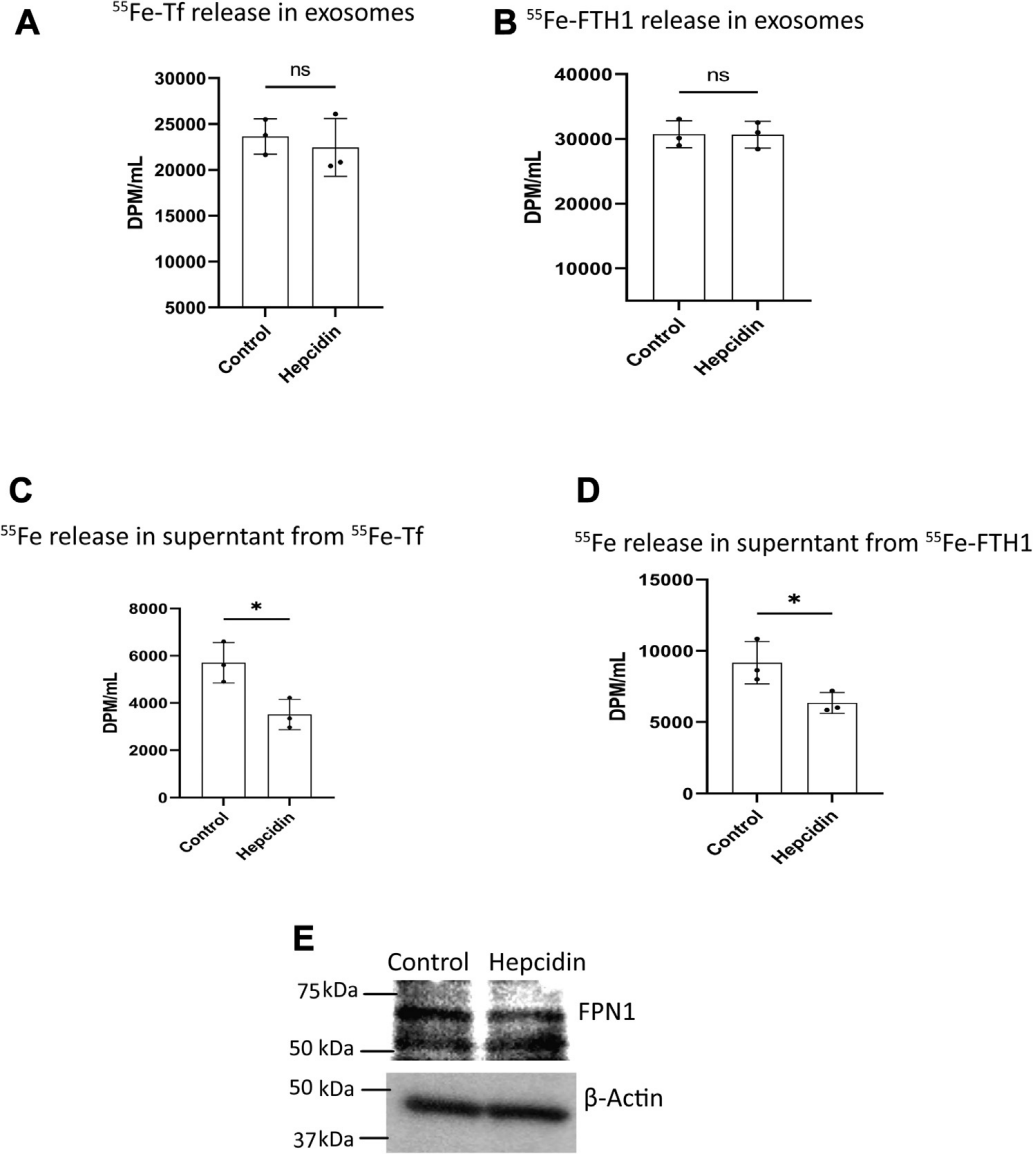
**Release of exosomes containing**^**55**^**Fe-Tf or**^**55**^**Fe-His-FTH1 is independent of hepcidin regulation in BBBECs.** ECs were grown on 0.4 μm pore size filters. ^55^Fe-Tf (1 mg/ml) or ^55^Fe-FTH1 (100 μg/ml) were added to the apical chamber and 1 μmol/L hepcidin was added to the basal chamber concurrently. Exosomes were isolated from the basal chamber media, and ^55^Fe activity was measured in exosomes and supernatant fractions of control and hepcidin treatment. *A* and *B*, illustrate that hepcidin has no effect on the exosomes release of ^55^Fe-Tf or ^55^Fe-HisFTH1 respectively. ns nonsignificant; *C* and *D*, demonstrate that hepcidin significantly reduces supernatant ^55^Fe transported from the ^55^Fe-Tf or ^55^Fe-HisFTH1 respectively. ^55^Fe activity was measured in DPM/ml. *E*, illustrates immunoblot analysis of FPN1 expression in control and hepcidin treatment. The experiment was performed in three independent replicates, data are shown as the mean ± S.D. Unpaired *t* test. ∗*p* < 0.05. All experiments were performed with three technical replicates in each biological replicate. BBBECs, blood–brain barrier endothelial cells; ECs, endothelial cells; FPN1, ferroportin1; FTH1, H-ferritin; Tf, transferrin.

Astrocytes release hepcidin to regulate iron release from BBBECs (22, 27). To determine whether hepcidin impacts the release of Tf- or FTH1-bound iron in exosomes, ECs were treated with hepcidin (1 μmol/L) in the basal chamber while simultaneously adding ^55^Fe-Tf or ^55^Fe-His-FTH1 to the apical chamber. ^55^Fe-Tf or ^55^Fe-His-FTH1 was added to the apical chamber in the absence of hepcidin in the basal chamber as a control. After 24 h, exosomes were isolated from the basal chamber media and measured ^55^Fe activity in exosomes and supernatant fractions. There was no significant difference in ^55^Fe activity in the exosomes between the hepcidin-treated and control groups (Fig. 5, *A* and *B*). However, when we determined the ^55^Fe levels in the supernatant (nonexosomal fraction) it was significantly reduced by hepcidin compared to the control (Fig. 5, *C* and *D*). To confirm that hepcidin reduced the release of ^55^Fe by inducing the degradation of FPN1, we added 1 μmol/L of hepcidin to the basal chamber, which resulted in 44% reduction (*p* < 0.05) of FPN1 levels compared to the control (Fig. 5*E*).

## Discussion

Our previous studies have shown that FTH1 is a major iron source to the oligodendrocytes (17) and BBBECs (23), and appears to be a significant iron source during brain development (39). The role of Tf in brain iron uptake is well-established (8, 40). In the current study, we investigated the transport of Tf, FTH1, and iron through BBBECs *via* exosomes and the status of iron on the release of exosomes. First, we confirmed that ECs release exosomes to the basal chamber. Previous studies showed that human and mice BBBECs release exosomes and are involved in the transport of proteins and miRNA (41, 42, 43, 44). However, this study is the first to demonstrate exosomes released from the hiPSCs-derived human ECs and to propose the concept that such exosomes may serve as a significant source of iron and iron transport for the brain. Therefore, we studied the impact of exosomes on Tf- and FTH1-bound iron transport in BBBECs. Exosomes isolated from media in the basal chamber contained significantly higher ^55^Fe-TF or ^55^Fe-His-FTH1 compared to the corresponding supernatant (nonexosome containing) fraction (Fig. 2, *A* and *B*). These results are consistent with ours and other studies that demonstrate that Tf- and FTH1-bound iron is transported to the brain through BBBECs *via* what appeared to be transcytosis (10, 11, 12, 45). However, to further clarify the apparent mechanism, we studied the consequences of the exosome inhibitor (GW4869) on the transport of Tf- or FTH1-bound iron. GW4869 significantly reduced the transport of the ^55^Fe-Tf and ^55^Fe-His-FTH1 in exosomes compared to the control, suggesting that much of the apparent transcytosis was mediated through exosomes.

Our recent studies in human BBBECs demonstrated that Tf and FTH1-bound iron is released from ECs as both free and protein-bound (23). The present study expands these findings to demonstrate that Tf and FTH1 deliver iron through exosomes. We also observed the ECs transported iron from Tf and FTH1 in a nonexosomal pathway. The nonprotein-bound iron release was regulated *via* FPN1/HEPH axis and regulated by hepcidin (23, 27, 46). Hepcidin is a hormone secreted by the liver, known to regulate cellular iron release, particularly by degrading the iron exporter protein FPN1 (28, 29, 47). In the brain, astrocytes have been shown to release hepcidin which similarly inhibits iron release from the BBBECs *via* degrading FPN1(27). In this study, hepcidin did not have any effect on exosome containing ^55^Fe-Tf– and ^55^Fe-His-FTH1–bound iron from the ECs, which is consistent with our data showing that hepcidin does not impact protein-bound iron transport in ECs and only effects the free iron release (23). Based on these data, we conclude that ECs transport the ^55^Fe-Tf– or ^55^ Fe-His-FTH1–bound iron in exosomes independently of hepcidin regulation. Moreover, the released exosomes contain significantly more Tf- and FTH1- bound iron than supernatant (nonexosome), indicating exosomes are a primary source of iron released by the BBBECs.

In the current study, we found that the release of ferritin and exosomes from the BBBECs is iron responsive which is in agreement with fibroblast cells isolated from normal lung tissue (38). Intracellular iron homeostasis is primarily achieved by posttranscriptional regulation of iron metabolic proteins by IRPs (48, 49). In high iron concentrations, IRPs are degraded, and the IRE in the 5′ UTR of FTH1 mRNA becomes unbound resulting in increased synthesis of ferritin protein (50). We also found that FAC treatment increases the release of ferritin within exosomes as well as CD63, a marker found on exosome membranes. CD63 and ferritin mRNA both have IREs in 5′ UTR, thus in cells treated with FAC, their protein synthesis is coordinately regulated, and CD63 specific exosomes are then released, enriched in FTH1, into the extracellular space (32). Our results are consistent with a report that FAC increases the release of ferritin in CD63^+^exosomes and the number of exosomes (38). In the current study, FAC treatment in BBBECs increases the expression of both CD63 and CD81 in cellular and released exosomes. In contrast, previous studies in fibroblast cells show that FAC increases only CD63, but not CD81. CD81 mRNA does not contain IRE sequences and thus its relationship to cellular iron status is not clear; although there is a report of a possible interaction between CD81 and the TfR2 (51). A novel finding in our study is that iron chelation with DFO results in a decreased release of total exosomes and exosome membrane protein (CD63 and CD81) expression. DFO can induce additional effects in cells, particularly induction of hypoxia. Thus, the impact of iron chelation on exosomal membrane proteins may be both indirect as well as direct. It should be noted, however, that hypoxia reportedly reduces the TEER values in the BBB model (52). In our study, we did not see any effect of DFO treatment on TEER values compared to the control or FAC treatments, suggesting hypoxia is an unlikely explanation for the effect on the exosome release. Moreover, the release of FTH1 and Tf in the exosomes is also significantly decreased compared to the iron loaded cells. Together, these results suggest that iron concentration in the BBBECs regulates the release of exosomes containing Tf and FTH1 into the brain.

In summary, our study demonstrates that BBBECs transport iron to the brain by exosome and nonexosome mechanisms that are regulated by the iron concentration in the ECs. Moreover, the exosomal fraction provides at least 2× more iron than nonexosomal fraction. Only the nonexosomal fraction is subject to regulation by hepcidin. This is the first demonstration that iron is transported into the brain *via* exosomes released by BBBECs. Furthermore, this novel exosome-mediated iron transport pathway could provide exciting opportunities to explore mechanisms for the delivery of therapeutic compounds as the iron delivery system to the brain. The lack of regulation of exosome iron delivery by hepcidin indicates the exosome delivered iron is not subject to manipulation by astrocyte inflammatory responses, but the amount of iron delivered *via* exosome is clearly regulated by the iron content of the ECs, consistent with our premise that iron uptake into the brain is regulated at the level of the ECs (23).

## Experimental procedures

### BBBECs derives from induced pluripotent stem cells

Human endothelial cells were differentiated from ATCC-DYS0100 hiPSCs as described previously (53, 54). hiPSCs were seeded onto a Matrigel-coated plate in E8 medium (Thermo Fisher Scientific# 05990) containing 10 μM ROCK inhibitor (Y-27632, R&D Systems; #1254) at a density of 18000 cells/cm^2^. The iPSCs differentiation was initiated by changing the E8 medium to E6 medium (Thermo Fisher Scientific A1516401) after 24 h of seeding. E6 medium was changed every 24 h, and cells were continued to E6 medium for up to 4 days. After 4 days, cells were switched to basal endothelial medium (hESFM) (Thermo Fisher Scientific #11111) supplemented with 10 nM bFGF (Fibroblast growth factor, Peprotech # 100–18B) and 10 μM RA (retinoic acid, Sigma #R2625) and 1% B27 (Thermo Fisher Scientific#17504–044). The medium was not changed for 48 h. After 48 h, cells were collected and replated onto transwell filters or cell culture plates coated with collagen IV and fibronectin. Twenty-four hours after replating, bFGF and RA were removed from the medium to induce the barrier phenotype.

### His-H-ferritin

Recombinant FTH1 was prepared as previously described (23). Briefly, wildtype human FTH1 containing a poly-His tag was subcloned into pET30a (+) to be produced in BL21 *Escherichia coli*. Isopropyl-β-D-thio-galactoside was used to induce expression. Following this, bacteria were lysed, and FTH1 protein was purified on a nickel column using standard techniques (GE Healthcare Bio-Sciences).

### ^55^Fe-labeling of FTH1 or Tf

The ^55^Fe labeling of Tf or FTH1 was performed as previously described (23). Briefly, ^55^Fe (PerkinElmer) was complexed with nitrilotriacetic acid, ferric chloride (FeCl_3_), and sodium bicarbonate (NaHCO_3_) at a ratio of 100 μl nitrilotriacetic acid: 6.7 μl FeCl_3_: 23.3 μl NaHCO3: 50 μCi ^55^FeCl_3_ to form the ^55^Fe-nitrilotriacetic acid complex. After complexing, ^55^Fe-nitrilotriacetic acid was subsequently incubated with apo-Tf (Sigma) or His-FTH1 for 30 min to allow for iron loading (12). Free iron was separated from the total complex using PD midiTrap-G25 columns following the manufacturer’s instructions (GE Healthcare Bio-Sciences).

### Exosomes isolation

The exosome isolation from the cell culture media was completed as described by Zhao *et al.*(55) by sequential centrifugation. Briefly, the basal chamber media were collected and centrifuged at (i) 300*g* for 10 min to remove the cell pellets, and (ii) the supernatant was centrifuged at 2000*g* for 10 min to remove the dead cell pellet. The resultant supernatant was centrifuged at 4000*g* for 30 min to remove the cell debris pellet. The resultant supernatant was collected in a 100K filter (Amicron Ultra -15, Centrifugal filters, Merck Millipore Ltd) to concentrate the media. The concentrated media were collected and centrifuged at 100,000*g* for 70 min (Beckman coulter, TLA 100.3). The supernatant was discarded, and the pellet was washed with PBS and again centrifuged at 100,000*g* for 70 min with the resultant exosome pellet used for downstream processing.

### Transport studies

The radiolabeled iron transport studies were described previously (23). Initially, the apical chamber of 12-well Transwell plates (Costar Transwell, 0.4 μm pore, Corning) was coated with collagen IV (Sigma) and fibronectin (Sigma) at a ratio of 5:4:1 of ddH_2_O, 1 mg/ml collagen IV, and 1 mg/ml fibronectin respectively. A total of 200 μl was used to coat plates 4 h at 37 °C. Following coating, the differentiated cells were replated onto the collagen coated plates. The basal chamber was filled with 1.5 ml of the same media. After allowing the cells to attach overnight at 37 °C, we changed the media in both chambers to hESFM, supplemented with 1% B27, but lacking bFGF and RA. Cells were incubated at 37 °C overnight to allow growth and tight junction formation, after which all transport studies were performed. Immediately before the experiment was performed, a complete media exchange was conducted by washing three times with 1 × DPBS to remove B27; 500 μl of serum-free media was added to the apical chamber, while 1.5 ml was added to the basal chamber. After media addition, transendothelial electrical resistance measurements were taken using an Epithelial Volt/Ohm Meter for transendothelial electrical resistance (TEER) (EVOM2, STX2, World Precision Instruments). Blank (media only) TEER readings were obtained and subtracted from all other TEER measurements. Across all experimental conditions, we report an average TEER value of 3800 ± 126 Ω × cm2. RITC-Dextran (70 kD, Sigma) was added at the beginning of the experiment to monitor tight junction formation and barrier permeability; GW4869 (20 μM, #D1692, Sigma) or 120 μCi of either the ^55^Fe-Tf (1 mg/ml) or ^55^Fe-His-FTH1 (100 μg/ml) complex was added to the apical chamber, and cells were incubated at 37 °C, 5% CO_2_. Hepcidin (Peptides International, PLP-4392-s) was added at 1 μM to the basal chamber. Finally, ^55^Fe activity was measured at either 24 or 48 h.

### Immunoblotting

Protein expression was detected *via* immunoblot as previously described (23). Briefly, the isolated exosomes were dissolved in a mixture of RIPA buffer (Sigma) and protease inhibitor cocktail (PIC, Sigma). Subsequently, total protein was quantified by bicinchoninic assay (Pierce), and 15 μg of exosomes and 25 μg cellular protein were loaded onto a 4 to 20% Criterion TGX Precast Protein Gel (Bio-Rad). Proteins were transferred onto PVDF membrane and probed for FTH1 (Cell Signaling Technology, 1:1000, 4393S), Tf (Abcam, 1:1000, ab82411), CD63 (Thermo Fisher Scientific; 1:1000, 10628D), CD81 (Cell Signaling Technology; 1:1000, 52,892), IRP2 (cell Signaling Technology;1:1000, 37,135), Ferroportin1 (Alpha diagnostic International, 1:1000, MTP-11S) Calnexin (cell Signaling Technology; 1:1000, 2679), or beta-actin (Sigma, 1:1000, A5441). Corresponding secondary antibody conjugated to HRP was used (1:5000, GE Amersham), and bands were visualized using ECL reagents (PerkinElmer) on an Amersham Imager 600 (GE Amersham).

### Nanoparticle tracking analysis

Exosomes quantification was performed using a NanoSight NS300 (Malvern Instruments Ltd, Malvern) previously (56). Briefly, exosome samples were diluted in 1 ml of particle free-water, and each sample was loaded by syringe pump into the NanoSight NS300 (Malvern Instruments Ltd, Malvern) set in scatter mode, and five 60-s videos were generated at 24.98 frames/sec. The size distribution and concentration of particles were calculated, and images were acquired using NanoSight software, version 3.2 (Malvern Instruments Ltd).

### Transmission electron microscopy

TEM was performed as previously described (57). Briefly, 10 μl of exosomes solution was placed on parafilm. Formvar-coated copper grids were then placed on top of the drops and incubated for 20 min. The copper grids were then incubated with a 4% solution of paraformaldehyde in 0.1 M PBS for 20 min, washed thrice with PBS for 1 min each, incubated with 1% glutaraldehyde in 0.1 M PBS for 5 min, washed with distilled water for 2 min, washed thrice with PBS for 2 min each, contrasted with 1% uranyl acetate for 20 s, and then observed by TEM (JEOL-1400).

### Statistics

All data were expressed as mean ± SD. Statistical analysis was carried out using GraphPad Prism 9. Student’s unpaired *t* test was used to compare between two groups. One-way ANOVA followed by Tukey’s post hoc analysis test was used to detect statistical significance (*p* < 0.05) between the multiple groups.

## Data availability

All the data analyzed and generated during this study are included in this article. All raw data contained in the manuscript are available from the corresponding author upon request.

## Supporting information

This article contains supporting information.

## Conflict of interest

The authors declare that they have no conflicts of interest with the contents of this article.
