# Supplementary figures and images for: Exosomes are involved in iron transport from human blood–brain barrier endothelial cells and are modified by endothelial cell iron status
Source: J Biol Chem. 2023 Jan 3;299(2):102868. doi: 10.1016/j.jbc.2022.102868 (PMC9929479; doi:10.1016/j.jbc.2022.102868)

Supplementary Figure 1

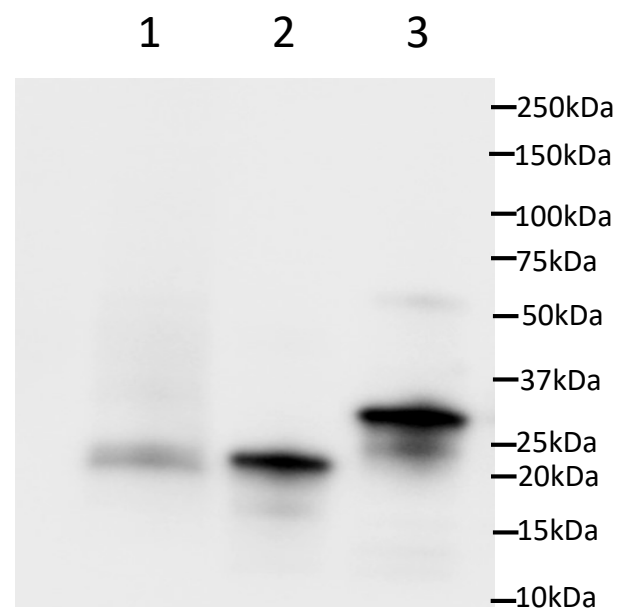

Supplement: Supplemental Figure S1 [file mmc1.pdf]
