# Supplementary material for: Exosomes are involved in iron transport from human blood–brain barrier endothelial cells and are modified by endothelial cell iron status
Source: J Biol Chem. 2023 Jan 3;299(2):102868. doi: 10.1016/j.jbc.2022.102868 (PMC9929479; doi:10.1016/j.jbc.2022.102868)
Supplement: Supplemental Figure Legend [file mmc2.docx]

**His-FTH1 (H-Ferritin) molecular weight distinguished from the cellular FTH1:** To this, we performed SDA-PAGE and immunoblot and probed with FTH1 antibody. Well 1, demonstrating the Endothelial cells endogenous FTH1, well 2, demonstrating the Human recombinant FTH1 procured from the Novus biological (NBC1-18548, Novus Biologicals, USA), well 3- demonstrating His tag FTH1produced from the E. Coli.
